# Draft Genome Sequence of *Methylobacterium* sp. ME121, Isolated from Soil as a Mixed Single Colony with *Kaistia* sp. 32K

**DOI:** 10.1128/genomeA.01005-15

**Published:** 2015-09-03

**Authors:** Shun Fujinami, Kiyoko Takeda-Yano, Takefumi Onodera, Katsuya Satoh, Tetsu Shimizu, Yuu Wakabayashi, Issay Narumi, Akira Nakamura, Masahiro Ito

**Affiliations:** a Bio-Nano Electronics Research Centre, Toyo University, Kawagoe, Saitama, Japan; b Department of Chemistry, College of Humanities and Sciences, Nihon University, Setagaya-ku, Tokyo, Japan; c Faculty of Agriculture, Tokyo University of Agriculture and Technology, Fuchu, Tokyo, Japan; d Cooperative Research Centre of Life Sciences, Kobe Gakuin University, Kobe, Hyogo, Japan; e Ion Beam Mutagenesis Research Group, Biotechnology and Medical Application Division, Quantum Beam Science Center, Japan Atomic Energy Agency, Takasaki, Gunma, Japan; f Faculty of Life and Environmental Sciences, University of Tsukuba, Ibaraki, Japan; g Graduate School of Life Sciences, Toyo University, Itakura-machi, Gunma, Japan; h Faculty of Life Sciences, Toyo University, Itakura-machi, Gunma, Japan

## Abstract

*Methylobacterium* sp. ME121 was isolated from soil as a mixed single colony with *Kaistia* sp. 32K, and its growth was enhanced by coculture. Here, we report the draft genome sequence of *Methylobacterium* sp. ME121, which may contribute to the study of the molecular mechanisms underlying this phenomenon.

## GENOME ANNOUNCEMENT

It has been suggested that a majority of environmental microorganisms are still uncultured, and some of them form symbiotic relationships with other organisms. For example, *Symbiobacterium thermophilum* was reported as a symbiotic bacterium requiring support of the associated *Geobacillus stearothermophilus* for growth (1). The genomic information of such symbiotic bacteria could be of use for studying the molecular mechanisms underlying microbial symbiosis (2).

*Methylobacterium* sp. ME121 was isolated from soil as a mixed single colony with *Kaistia* sp. 32K during our screening of l-glucose-utilizing microorganisms (3), and its growth was enhanced by coculture. It was expected that genomic analysis of this bacterium would provide novel information on coculture-dependent growth enhancement. *Methylobacterium* sp. ME121 appeared to be most closely related to *M. radiotolerans* based on the 16S rRNA gene sequence identity.

The draft genome sequence of *Methylobacterium* sp. ME121 is 7,096,979 bp in total length and comprises 197 large contigs (>500 bp) and was obtained with the Roche GS Junior and assembled using the GS *de novo* assembler version 2.7. Automatic annotation was performed using the Microbial Genome Annotation Pipeline (4), which predicted a total of 6,676 protein-coding genes. The product names of the predicted protein-coding genes were revised manually. tRNA detection was performed using the tRNA scan software (5), which predicted a total of 56 tRNAs.

*Methylobacterium* species generally live on plant surfaces and assimilate methanol emitted by plants. The genome sequences of *M. aquaticum* and *M. radiotolerans* were analyzed, and the genes involved in methylotrophy were identified (6, 7). The annotation of the draft genome sequence shows that *Methylobacterium* sp. ME121 has some genes that encode putative methanol/ethanol family PQQ-dependent dehydrogenases involved in methylotrophy. Some unknown factor (e.g., methanol) provided by the coculture may contribute to increase the growth of *Methylobacterium* sp. ME121. Future study will identify such a factor, and it would serve to clarify the molecular mechanisms of coculture-dependent growth enhancement.

### Nucleotide sequence accession numbers. 

The draft genome sequence of *Methylobacterium* sp. ME121 was deposited at DDBJ/EMBL/GenBank under the accession number BBUX00000000. The version described in this paper is the first version, BBUX00000000.1.
